# Should fast breathing pneumonia cases be treated with antibiotics? The scientific rationale for revisiting management in Low and Middle income countries

**DOI:** 10.1016/j.ijid.2019.05.035

**Published:** 2019-08

**Authors:** Fyezah Jehan, Imran Nisar, Salima Kerai, Nick Brown, Gwen Ambler, Anita K.M. Zaidi

**Affiliations:** a Department of Paediatrics and Child Health, Aga Khan University, Pakistan; b International Maternal and Child Health (IMCH), Department of Women’s and Children’s Health, Uppsala University, Uppsala, Sweden; c Department of Paediatrics, Länssjukhuset Gävle-Sandviken, Gävle, Sweden; d PATH, Seattle, WA, United States

**Keywords:** Non inferiority, Placebo, Fast breathing pneumonia

## Abstract

**Background:**

Pneumonia is the largest single contributor to child mortality and the problem is more acute in low and middle income countries. The World Health Organization (WHO) currently recommends oral antibiotic treatment for all children with fast breathing pneumonia without danger signs. It is, however, widely acknowledged that most such infections are viral and self-limiting and that the evidence for the guidance is weak.

**Rationale:**

Overuse of antibiotics exposes children to adverse events, increases cost for families, burdens already stretched health care resources and may contribute to development of antibiotic resistance.

**Conclusion:**

There is equipoise regarding utility of antibiotic in case of fast breathing pneumonia and no high quality trial evidence exists. This paper provides further information behind the rationale for conducting non-inferiority trials to test the hypothesis that antibiotics may not be necessary for children with fast breathing as the sole symptomatology.

## Highlights

• World Health Organization (WHO) recommends oral antibiotic treatment for all children with fast breathing pneumonia.
• However evidence for the guidance is weak and infections are often viral and self-limiting.
• Further information regarding the true rationale for conducting non-inferiority trials to test the hypothesis that antibiotics may not be necessary for children with fast breathing as the sole symptomatology.

## Background

Pneumonia is the major cause of post-neonatal mortality in children under five years of age, contributing annually to over a million deaths, of which two thirds occur in low and middle income countries (LMIC) (Rudan et al., 2008). The World Health Organisation (WHO) uses clinical syndromal definitions according to severity. The WHO currently recommends antibiotic treatment for children aged 2–59 months with suspected lower respiratory tract infection to cover the possibility of bacterial infections (World Health Organization, 2014).

Treatment allocation is made according to the severity of illness which is based on clinical criteria made by observation. Until 2014, classification was made into four categories: no pneumonia, mild (essentially fast breathing alone), severe (with chest indrawing with or without fast breathing) and very severe a definition requiring additional danger signs. The first two categories are felt appropriate for primary health care and home management with oral antibiotics: the third requires secondary centre referral, monitoring and parenteral antibiotic use.

The broad recommendation for children with ‘fast breathing pneumonia without danger signs’ is based on the assumption that a proportion of children in the most resource limited settings will not have the means to re-consult should the picture change. However, evidence for the guidance is weak and infections are often viral and self-limiting. This has generated substantial debate among experts (Hazir et al., 2011, Awasthi et al., 2008). There is equipoise regarding utility of antibiotics in fast breathing pneumonia and WHO has repeatedly identified a need for research for providing high quality evidence regarding appropriate management of community-acquired pneumonia (CAP). In 2014 a Cochrane review investigated the existing evidence comparing antibiotic to no antibiotic treatment for fast breathing pneumonia. The study found a lack of research in this area and concluded that “we do not currently have evidence to support or challenge the continued use of antibiotics for the treatment of non-severe (reclassified fast breathing) pneumonia, as suggested by WHO guidelines” (Lassi et al., 2014).

Moreover, increasing global coverage of effective vaccines (Pneumococcal and *Haemophilus influenza type b*) against the two major bacterial causes of childhood pneumonia in GAVI-eligible countries, including Pakistan. The epidemiology is changing and, though non-vaccine serotypes may become more prevalent, data to date suggest that these infections are likely to become less important contributors to pneumonia morbidity (Levine et al., 2006, Cowgill et al., 2006) and that the proportion of viral cases likely to increase. The changing epidemiology of the disease, therefore, requires a re-evaluation of practice related to use of antibiotics.

This review presents the scientific rationale of performing non-inferiority studies in children with fast breathing pneumonia, comparing amoxicillin (control) to a placebo intervention. There is such a trial underway in Pakistan, the results of which should provide evidence to support or refute current WHO guidance (Jehan et al., 2016).

## Main text

### Scientific rationale

Withholding WHO recommended antibiotic treatment has a sound scientific rationale essentially because WHO-defined “fast breathing pneumonia” is a misclassification in the majority of cases (Izadnegahdar et al., 2013). Tabish et al in a study of 1848 children with fast breathing in Pakistan found that only 14% had radiological evidence of pneumonia, while the rest had either normal chest X-rays (82%) or bronchiolitis (4%) (Hazir et al., 2006). Previous studies have shown a high rate of resolution without treatment and there is evidence that amoxicillin has only partial efficacy in resolving this sign. In some settings, up to 65% of non-severe pneumonia is viral in aetiology with a bacterial viral co-infection in about 30% (Grant et al., 2009, Ruuskanen et al., 2011). Spontaneous remissions are frequent that may render antibiotics partly or completely ineffective. Current management guidelines prioritise sensitivity over specificity, resulting in widespread use of antibiotics when they are not needed (Izadnegahdar et al., 2013, Qazi and Were, 2015, English and Scott, 2008, Maitland, 2014).

A fundamental principle of medical practice is to “do no harm.” By prescribing antibiotics to children that do not need them, there are potential risks and negative consequences at both the individual and population levels. Risks to children include an increased exposure to adverse events associated with antibiotics, which may be both unpleasant and dangerous. Moreover, early life exposure to antibiotics has shown to increase the risk of allergic disease in childhood (Kuo et al., 2013). There is also a potential long-term deleterious effect on the native gut microbiota which may be altered immune processing resulting in long-term risk of subsequent infections (Kristinsson, 1997, Uzuner et al., 2007, Woolfson et al., 1997, Murni et al., 2014, Rizal et al., 2010, Jonathan and Stoltenberg, 2012, Mauri and D’Agostino, 2017). At the population level, indiscriminate/injudicious use of antibiotics has increased risk of antimicrobial resistance (Kristinsson, 1997, Uzuner et al., 2007, Woolfson et al., 1997), resulting in the rise of antibiotic-resistant strains of bacteria and the need to use more expensive alternatives with greater risk of adverse events (Murni et al., 2014). Good antibiotic stewardship is increasingly important for amoxicillin to remain a long-term solution for treating childhood pneumonia worldwide (Rizal et al., 2010, Jonathan and Stoltenberg, 2012).

### Feasibility of non-inferiority placebo controlled design

Testing a placebo intervention against an active control requires a non-inferiority trial, which works on the basis of the margin of failure being within a margin deemed a priori to be acceptable. Employing a non-inferiority trial is much more complex in the design, implementation and analysis (Mauri and D’Agostino, 2017). It is impossible to establish non-inferiority of no antibiotics to existing treatment without undertaking a robustly performed and adequately powered randomized controlled trial with low attrition and per-protocol analysis (Lewis et al., 2002). The most important aspect of such placebo-controlled trials is patient safety and it is fundamental to follow patients in the first 72–96 h after recruitment to guarantee safety. If this is made in a site with HDSS this might be facilitated though is not compulsory. Moreover, these trials must be designed in such a way that continued surveillance and easy re-access to health facilities is feasible and that rescue treatment introduction possible in case of deterioration and failure of expected resolution. (Lewis et al., 2002). Such trials should be blinded and randomized to reduce potential bias and enhance the quality and generalizability of study results, considered the “most important design techniques for avoiding bias in clinical trials” (International Conference on Harmonisation EEWG, 1999). In addition, these trials should be scrutinized for protocol deviations or violations and failures by external oversight by both a data safety monitoring board and trial steering committee. Furthermore, the participant exposure to placebo should be made for short duration and it is necessary to ensure careful and regular monitoring to detect early treatment failure signs through a robust safety net. Such active surveillance could result in relatively better standard of care in comparison to cases outside a trial setting, so the risk of harm may be further reduced (Lewis et al., 2002).

### Ethical issues

Ethical analysis permits the use of placebo where the obligation is to determine the efficacy or safety of an intervention (in this case absence of treatment) provided there are sound methodological reasons and justification for using placebo and patients who receive placebo or no treatment will not be subject to any risk of serious harm (Millum and Grady, 2013), or subjects may benefit from being in the placebo group (van der Graaf, 2015). Placebo-controlled trials are justified when there is genuine equipoise and participants are not exposed to harm. There must be close clinical supervision, and a position of genuine informed consent (Lewis et al., 2002, van der Graaf, 2015). In these situations, international ethical standards in research allow for placebo to be used even if a known intervention exists. The Declaration of Helsinki discusses the use of placebo and notes that it may be used “where for compelling and scientifically sound methodological reasons the use of any intervention less effective than the best proven one, the use of placebo, or no intervention is necessary to determine the efficacy or safety of an intervention” (World Medical Association, 2013).

Most children require treatment with oral antibiotic solutions, which cost more and require refrigeration. This places a financial burden on families who bear these expenses out of their pocket and it also puts a strain on already under-resourced programmes in low-income settings. Dispersible Amoxicillin tablets are available through UNICEF, at a lower cost to the consumer, but availability is still non-uniform.

## Conclusion

There are sound biological and societal reasons for revisiting the management of fast breathing pneumonia in children. Equipoise in treatment, low risk of harm and the potential benefits of rationing antibiotic use are strong justifications for a non-inferiority trial.

## Ethics approval and consent to participate

Not applicable.

## Consent for publication

Not applicable.

## Availability of data and material

Not applicable.

## Competing interests

The authors declare that they have no competing interests.

## Funding

This manuscript is based on RETAPP trial which is jointly funded by the MRC-Wellcome-DFID through the Joint Global Health Trials, [grant number MR/L004283/1] and the Bill & Melinda Gates Foundation [grant number OPP1158281]. Fyezah Jehan and Imran Nisar received funding from the National Institute of Health’s Fogarty International Centre [Grant Number 1 D43 TW007585-01].

## Authors’ contributions

FJ, AZ, IN conceived the idea. FJ, IN and SK were responsible for drafting the manuscript. FJ, SK, NB and GA revised the manuscript critically for important intellectual content. All authors reviewed and approved the final version.
